# *M. caprae* in northern Italy: a comprehensive analysis through whole-genome sequencing on the genetic variability in bovine herds

**DOI:** 10.1186/s13567-025-01585-x

**Published:** 2025-08-07

**Authors:** Cristina Bertasio, Valentina Carta, Giovanni Parisio, Mariagrazia Zanoni, Marco Tamba, Laura Mazzera, Erika Scaltriti, Maria Lodovica Pacciarini, Giovanni Loris Alborali, Daniel Polzer, Annette Nigsch, Romana Steinparzer, Maria Beatrice Boniotti

**Affiliations:** 1 National Reference Centre for Bovine Tuberculosis, Istituto Zooprofilattico Sperimentale Della Lombardia E Dell’Emilia Romagna, Brescia, Italy; 2 Risk Analysis and Genomic Epidemiology Unit, Istituto Zooprofilattico Sperimentale Della Lombardia E Dell’Emilia Romagna, Parma, Italy; 3 Austrian Agency for Health and Food Safety (AGES), Department for Animal Health, Mödling, Austria

**Keywords:** *Mycobacterium caprae*, whole genome sequencing, SNP, VNTR-MIRU genotypes, bovine tuberculosis, phylogeny, spoligotypes

## Abstract

*Mycobacterium* (*M*.) *bovis* and *M. caprae* are the causative agents of bovine tuberculosis (bTB), which is still a concern due to its health implications and economic impact. Although *M. caprae* is less prevalent than *M. bovis* among bovine tuberculosis cases, it has a significant impact on animal health especially in Europe, where it has been isolated from a range of hosts. Starting from spoligotyping and the MIRU-VNTR profile specifically associated with the Lechtal subgroup we decided to process a selection of Italian *M. caprae* isolates using whole genome sequencing (WGS) to define the phylogenetic relationships between isolates and deepen the understanding of this public health issue. In this study, 20 outbreaks of bovine tuberculosis caused by *M. caprae* in northern Italy were retrospectively investigated by interpreting and validating WGS results with available epidemiological information. Genomes of 34 Italian strains, with a known and traditionally typed genotype, isolated between 2001 and 2022 from 21 bovine farms, with 12 isolates from Austria, were analyzed focusing on single nucleotide polymorphisms (SNP) to derive evolutionary relationships, pairing with the tracing of infections, suggested by the epidemiological contacts. The results outline possible connections between outbreaks, which caused the spread of the infection, obtaining two clusters differing by 6–16 SNP pairwise, overlapping with the territory distribution of the herds between two different Italian regions. Epidemiological information and phylogeny revealed that *M. caprae* was probably introduced in northern Italy from Austria and/or Germany, through separate and independent events for the two Italian clusters. The complex approach of integrating WGS data with epidemiological information proved to be useful in delineating likely transmission chains and identifying the possible sources of infection, showing how NGS is a powerful tool to support epidemiological investigations.

**Supplementary Information:**

The online version contains supplementary material available at 10.1186/s13567-025-01585-x.

## Introduction

Mammalian tuberculosis (TB) is an important zoonosis with serious implications for livestock and public health. Although it is caused mainly by *Mycobacterium bovis* (*M. bovis)*, bovine tuberculosis (bTB) can also be attributed to *Mycobacterium caprae*, a member of the *Mycobacterium tuberculosis* complex (MTBC), which represents an emerging pathogen with a significant impact on animal health, mainly across Europe [1–3].

Initially identified as primarily affecting goats [4], hence its name, *M. caprae* has been reported in a range of other domestic and wild animals, including cattle, pigs, bisons, deer, foxes, grey wolves, sheep and wild boar [5–11], and even in humans [12], reflecting its zoonotic potential.

The epidemiology of *M. caprae*, as well as *M. bovis*, is particularly challenging to trace because of its ability to infect multiple hosts and its presence in wildlife, that can act as reservoir, limiting the effectiveness of efforts to prevent the entry of infection on farms [13].

Worldwide, *M. caprae* has been detected and reported more frequently in the last decade, thanks to the increased use of spoligotyping, even from those countries where it was thought to be absent [3], like in North Africa with reported cases in Tunisia [14], Algeria [15] and Morocco [16], and in Asia with cases in China [17], Japan [18] and more rarely in other countries like Perù [19], Australia [20], United States [21] and Canada [12].

Particularly, the distribution of *M. caprae* is very noted in Europe, where it has been identified in several countries, including Germany [1, 7], Austria [22, 23], Italy [24], France [5], Spain [2, 25], Portugal [25], Slovenia [1], Czech Republic [10], Croatia [26], Greece [27], Bulgaria [28], The Netherlands [29] and Poland [30].

In Italy, a bTB eradication program has been active since 1995 and, in 2024, 13 regions including Lombardy and Emilia-Romagna regions and 16 provinces have been declared disease-free, according to European Union regulation.

Detecting epidemiological links is crucial for the control and the eradication of bTB: identifying where the infection originated and tracing how it spread through the territory helps experts gather evidence to plan interventions to stop the disease from spreading further. Analyzing genetic differences between strains from different outbreaks could be very important to understand the spread of the infection and also be crucial for improving eradication plans for tuberculosis in livestock. The epidemiology of tuberculosis can be studied not only with classic epidemiological investigations, but also with molecular biology techniques that allow strain characterization. Phylogeny and traditional epidemiology can interact and integrate in order to support correlation hypotheses.

Traditionally, epidemiological studies of bTB have been relying on genotyping techniques that analyse small genome fragments, like spoligotyping and mycobacterial interspersed repeat unit-Variable number of tandem repeats (MIRU-VNTR).

Since the 2000s, the Italian National Reference Center for Bovine Tuberculosis (NRC-TB) in IZSLER (Brescia, Italy) has been using classical methods to genotype *M. bovis*/*M. caprae* isolates, with both microbiological [31] and molecular assays [32]. This activity allowed to build a national database, named ITAN-TB, which counts more than 9300 samples associated with genotype, host and herd of origin: this information is used as a supportive tool to the epidemiological investigations, conducted within the Italian eradication program, providing also the first information on the genetic structure of the MTBC population circulating in Italy. In ITAN-TB, about 8% of outbreaks are typed as *M. caprae* up to 2020 [33]. They have been associated to many species, both belonging to livestock (bovine, buffalos and sheep), wildlife (red deer, wild boar and black swine) and unusual hosts like humans, Guinea pigs and macaques (data not published).

Loci-based approaches are useful for large-scale studies but can lead to homoplasies and insufficient resolution in studying local transmission events; they lack sufficient discriminative power to confirm recent individual transmission or distinguish remotely related MTBC strains, belonging to the same genotype. Indeed, the polymorphic sequences targeted represent a minimal part of the genome (less than 1%) of *M. bovis*/*M. caprae* and have a limited intrinsic differentiation capacity [34].

Domogalla et al. [35] identified an additional differentiation marker of *M. caprae* strains in the variability of the RD4 region, which enabled the discrimination of different genotypes associated with specific geographical distributions within the Alps. The authors examined the genetic diversity of *M. caprae* isolated from the Alpine region of Bavaria (Germany) focusing on the RD4 locus. The study identified three distinct RD4 variants, named Lechtal, Allgäu and Karwendel. When compared to the Allgäu subgroup, Karwendel and Lechtal subgroups are characterized by a 5 kb and a 38 kb deletion, respectively. These genetic differences reflect the phylogeographic distribution of *M. caprae* in the region and could influence transmission tracking and spread of this pathogen in both wildlife and livestock populations [36].

The use of whole genome sequencing (WGS) has significantly advanced the study of microbial populations, including mycobacteria, providing detailed insights into their genetic variations and transmission dynamics, offering a much higher resolution for cluster discrimination, resulting in an increased use of it in bTB outbreak investigations and the number of studies on this topic [33, 34, 37–40]. With the decline of WGS costs, its application to bTB control programs is desirable and highly recommended [41], since it might powerfully advance efforts in the future.

In the present study, we used WGS to characterize *M. caprae* strains isolated from different bovine herds of northern Italy between 2001 and 2022, with an identical/similar traditional genotype, obtained by the combination of spoligotype and MIRU-VNTR, suggesting a shared infection route. Pairwise nucleotide distances between samples were obtained by analysing WGS data. We used these data to investigate the possible transmission of the infection among and within herds, in international and national contexts. To the best of our knowledge, this is the first study conducted on *M. caprae* outbreaks in Italy through a WGS approach, able to fill some gaps left by the classical epidemiological investigation with objective data on the genomes. Furthermore, for the first time, starting from the Italian scenario, we tried to address cluster definition criteria based on SNP thresholds, hence defining the minimum number of SNP differences supported by epidemiological evidence.

## Materials and methods

### *Mycobacterium caprae* in ITAN-TB database

Mycobacteria of the MTBC were identified by PCR/RFLP of the gyrB gene, as reported by Boniotti et al. [42], and by two high resolution melting (HRM) assays targeting the gyrB gene, as recently described by Scaltriti et al. [33]. Since 2008, *M. bovis*/*M. caprae* strains were typed systematically by spoligotyping [43] and by VNTR-MIRU with ETRA-E [44] and 7 additional VNTR/MIRU markers selected in the study of Boniotti et al. [45], obtaining more accurate genetic profiles. General information such as sampling date, location and species was recorded for each sample, along with genotypic data; all this information constitutes the database of the NRC-TB, called ITAN-TB, which was used for sample selection. Finally, since 2022, the NRC-TB has begun using whole-genome sequencing in specific cases, including the data in its database.

### Sample selection

**Figure 1:**
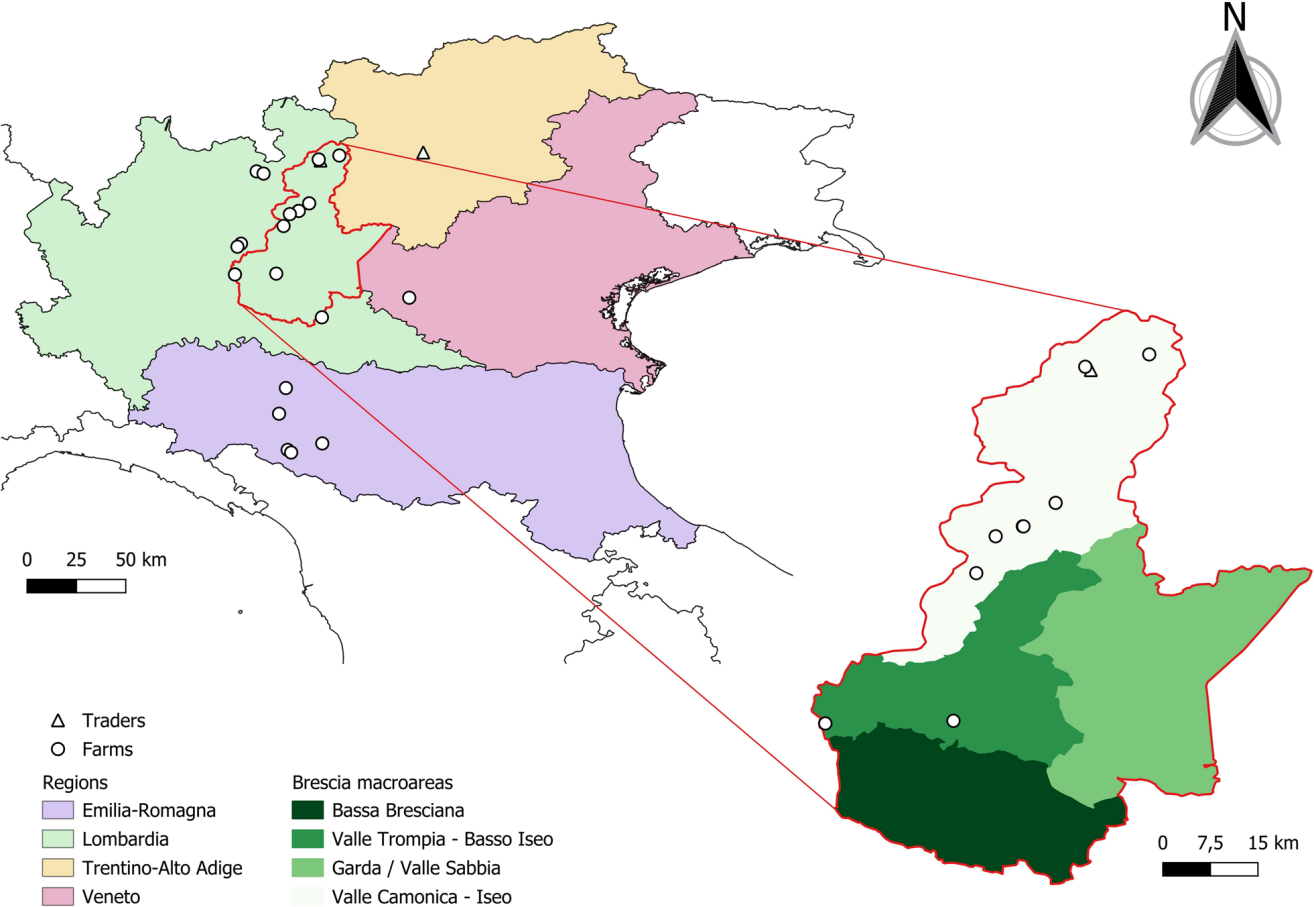
**Geographical location of *M. caprae* isolates of the dataset.** The borders of the province of Brescia are marked with a red line and the Brescia macroareas are reported on the right.

This retrospective study focused on 21 bovine farms in northern Italy where bTB has been reported since 2001 and continued to spread within herds located in the same area in subsequent years. The criteria for the inclusion were the geographic location, the spoligotype, the MIRU-VNTR profile, and the availability of the frozen stock in our laboratory. Sample selection for this study was performed exploiting the ITAN-TB database, focusing on *M. caprae* strains with SB0418 spoligotype, a MIRU-VNTR profile of 53523853434 (markers’ order: ETRA, ETRB, ETRC, ETRD, ETRE, Qub11a, Qub11b, Qub26, Qub1895, Qub15, MIRU26), and isolated from bovine herds in a restricted geographical area of northern Italy (Lombardy and Emilia-Romagna regions) over the period 2001–2022 (Additional file 1 and Figure 1). The marker Qub3232 was not considered because of its high variability in *M. caprae* genomes, as reported by Kremer et al. [46] and Duarte et al. [47]. A total of 34 isolates were selected. One isolate (ID 6627–2002), despite having a different MIRU-VNTR profile (allele 4 instead of allele 5 for ETRA), was still included in the dataset because of its geographical location and epidemiological connection with other herds. Additionally, an external strain isolated from a red deer in 2011 in the same geographical area and previously described by Chiari et al. [48], with the same genotype as in bovine samples, was included. It is important to note that each selected sample referred to one animal (1 sample = 1 animal), except for the two samples with ID 280428-4-2012 and 280428-13-2012 that derived from the same cattle.

### Epidemiological investigations

An outbreak investigation was performed on all the farms involved, to identify epidemiological links with other farms, considering animal and personnel movements, trading routes and possible relationships among the farmers. This activity was facilitated by the fact that in Italy all farms are registered with a unique code, all animals are individually and permanently identified with an ear tag and all their movements are recorded in the National Cattle Database [49].

### Sample preparation and DNA extraction

All isolates, stored frozen in tryptic soy broth with 10% glycerol in vials at −20 °C, were thawed and cultured on Stonebrink medium (prepared as solid slants in screw-cap tubes) in a BLS3 laboratory. Colonies were diluted in 200 μL Middlebrook 7H9 medium, reaching a 4 McFarland concentration. They were then incubated at 96°C for 20 min (1000 rpm) and sonicated for 15 min at 35 kHz at RT. Extraction was performed with a NucleoSpin Tissue kit (Machery-Naghel, Düren, Germany) according to manufacturer instructions. The concentration of the obtained DNA was assessed using the QuantiFluor^®^ ONE dsDNA System with the Quantus Fluorometer (Promega, Fitchburg, USA).

### Database assembly

To perform analysis, we built a dataset merging sequences obtained from the SRA archive, sequences kindly provided by AGES and sequences obtained from Italian samples.

Following research on the SRA database (09/04/2025 keywords used: “*Mycobacterium caprae*” and “*Mycobacterium tuberculosis* variant *caprae*”), a total of 468 sequences were identified. We kept only dated and geographically located sequences obtained from Illumina paired-end sequencing, reducing the selection to 459 sequences. SRA samples were distributed temporally and geographically, covering the period from 2002 to 2024 and originating from 12 different countries worldwide. These samples also came from different animal species, reflecting the typical host diversity of *M. caprae*.

Starting from the “General dataset”, which includes SRA sequences, AGES sequences and Italian sequences, we built three different datasets for phylogenetic analysis. The first, called “European dataset”, comprised all the SB0418 sequences, including 152 sequences isolated from Germany (62), Bulgaria (28), Italy (34), Spain (7) and Austria (12). The second dataset contained only sequences belonging to the “Lechtal subgroup” and comprised sequences from Germany (23), Spain (1), Italy (34) and Austria (12): this dataset was used to perform MJN analysis. The third dataset consisted solely of Italian sequences and was used to evaluate the most appropriate SNP threshold for the Italian scenario. All samples and the dataset used for this study are summarized in Additional file 2.

### Whole genome sequencing and SNP analysis

Genomic libraries were prepared using Illumina DNA Prep, (M) Tagmentation Kit (Illumina, San Diego, USA) and WGS was performed on either Miseq or Miniseq systems (Illumina, San Diego, USA) generating 2 × 150 bp paired-end reads.

All the reads in the dataset were checked for quality with the FastQC tool (v.0.12.1 [50]), and for contaminations with Kraken2 software [51]. Low quality reads were filtered out with Trimmomatic [52] (v.0.39) and high-quality reads obtained were used as input for SNP analysis.

To correctly compare Italian isolates within the international framework, we performed an SNP analysis using the “European dataset”, which comprised *M. caprae* isolates sequenced in this study, along with both a selection of representative SB0418 genomes from public databases and raw reads of 12 Austrian strains from red deer, provided by the Austrian Agency for Health and Food Safety GmbH (AGES). These 12 samples were collected during the period 2011–2021 and represent a subsample of all isolates gathered in the framework of routine monitoring. This extensive activity is being carried out in the regions affected by *M. caprae* in red deer in the western states of Austria, namely Tyrol and Vorarlberg, since 2008 and is still ongoing [22]. Each selected sample refers to one animal (Additional file 2).

The SNP analysis was carried out from trimmed reads with the MTBseq pipeline [53] (v.1.0.3) using the MTBC ancestral genome called “MTBC0”, recently described by Harrison et al. [54], as reference for the mapping, and filtering sequences for repetitive regions [53] and resistance associated genes [55].

Six *M. bovis* strains were used as the outgroup. A subset of this analysis was also used to generate Median-Joining Networks (MJ networks) using the software PopArt [56].

A second analysis based on a sub-dataset of only *M. caprae* isolates sequenced in this study was also performed using the MTBseq pipeline with the genome of *Mycobacterium caprae* subtype Allgäu (NCBI assembly number GCF_001941665.1) as reference for mapping. The TBgroups part of the pipeline was run, both leaving the threshold to the standard setting (12 SNP) and changing it down to 5 SNP settings.

The similarity between the considered samples was evaluated both considering the number of pairwise-SNP starting from the SNP matrix and through the phylogenetic analysis, which was conducted with a Maximum Likelihood tree with a bootstrap of 1000 via IqTree2 [57], successively visualized with FigTree. Furthermore, samples were inspected in IGV software to identify the genetic variations in the RD4 region to define the so-called “Allgäu”, “Karwendel” or “Lechtal” subgroups, according to Domogalla et al. [35]. Moreover, raw reads were analyzed with SpoTyping 2.1 tool [58] and the results were submitted on the *Mycobacterium bovis* Spoligotype Database [59] platform to confirm the SB0418 Spoligotype.

All Austrian sequences generated by AGES have been deposited at the SRA archive and are available under the accession numbers from SRR31790111 to SRR31790122.

All sequence data generated in this study have been deposited at the SRA archive and are available under the accession numbers from SRR29410367 to SRR29410390.

## Results

### *Mycobacterium caprae* in ITAN-TB database

Since 2000, 175 different *M. caprae* genotypes have been identified in Italy, particularly 22 different spoligotypes associated with 168 different MIRU-VNTR profiles. Among these, 125 genotypes are typed as SB0418. Up to 2025, the Italian National Reference Center for Bovine Tuberculosis collected a total of 566 samples. Of these, 76.8% were typed as SB0418, with 14.5% belonging to the selected MIRU-VNTR profile. Despite SB0418 samples being distributed across both northern and southern Italy, the samples associated with the genotype analyzed in this study are predominantly from the norther part of the country: 92% (58 out of 63) were collected from northern farms, with representation across all the provinces considered. Only five out of the 63 samples originated from southern Italy, and notably, two of those were from animals bearing Austrian ear tags. The temporal distribution of these genotypes shows its first identification in Italy in 2009, with peaks in 2012, 2018, 2019 and 2022.

### Epidemiological survey

The epidemiological investigation conducted allowed the collection of important information that clarifies commercial and non-commercial relationships and connections between the farms involved. Specifically, the investigation highlighted the purchase of cattle from a collection centre in Tyrol (Austria) and/or Germany. A collection centre is a structure where animals are kept temporarily before being acquired and/or moved to the purchasing farm. With a halfway step in Trentino Alto Adige, the infection spread among the Italian farms, in Lombardy and Emilia-Romagna, following independent routes.

**Figure 2:**
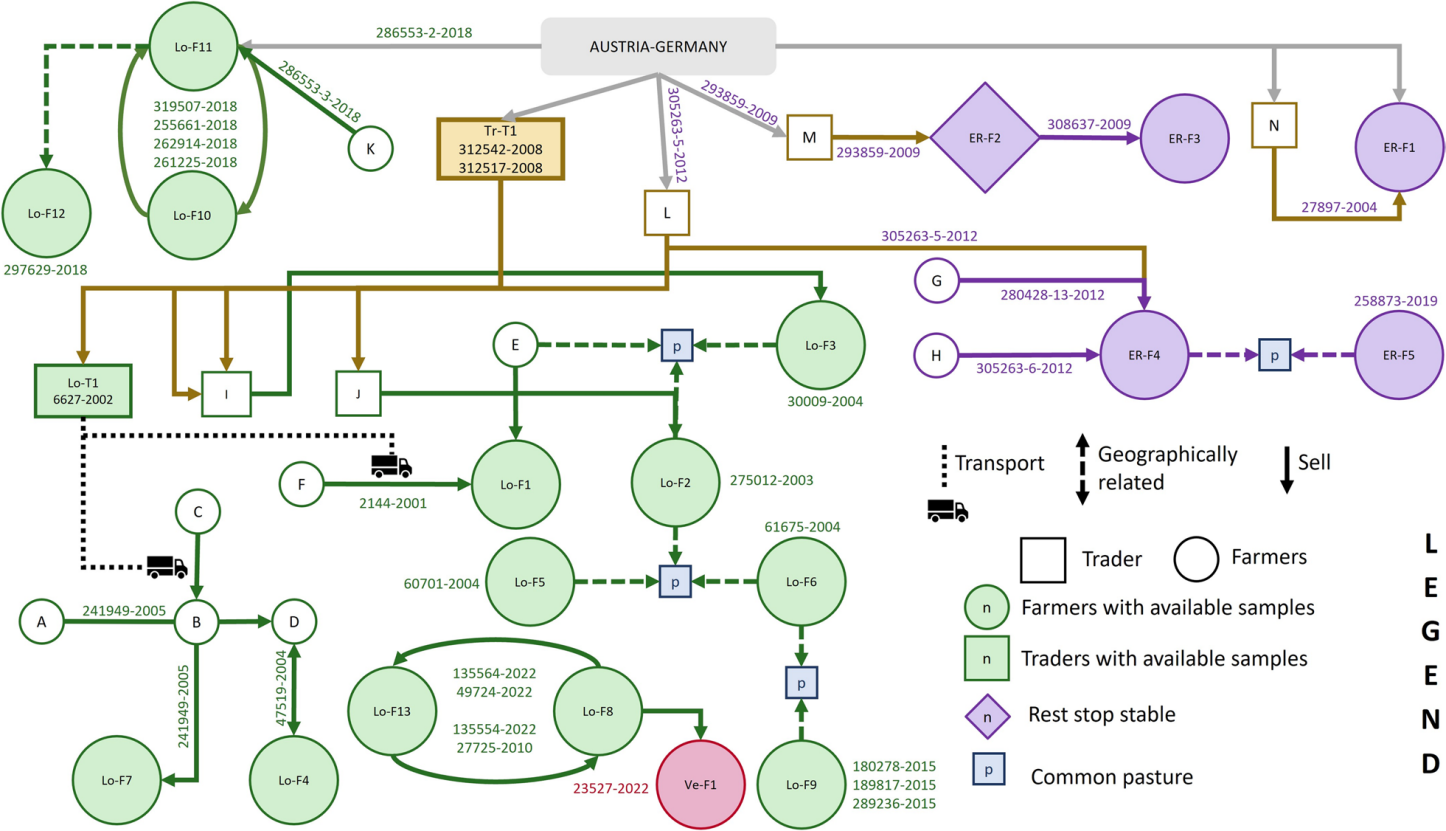
**Graphical representation of the connections and relationships among the bovine farms/traders investigated in this work. **Farms and traders are colour coded: green for Lombardy region, violet for Emilia-Romagna region, ochre for Trentino region and pink for the only Veneto farm. Filled shapes indicate farms from where at least one cattle was sampled, while empty shapes indicate farms epidemiologically involved in the network of exchanges but not sampled. Animals were purchased from abroad by some traders of Trentino Alto Adige region (Tr-T1, L, M, N) and then sold to three sellers of Val Camonica, Lombardy (Lo-T1, I, J), that supplied nearby farms, or directly to the Emilian farms. Sometimes animals were bought by the Italian farmers straight from Austria/Germany (Farm Lo-F11 and ER-F1).

As shown in Figure 2 (and explained in Additional file 3), the epidemiological investigation suggested a complex network of connections between farms, mainly involving trading routes, exchange of animals, common pastures and geographical proximity.

The survey revealed a shared trading route linking the considered outbreaks, identified as the most likely source of infection for both the Lombard and Emilian farms, without highlighting any evident direct connection between the outbreaks in the two regions.

First, the epidemiological survey allowed the identification and classification of all the farms involved: 19 (Lo-F1 to F13 and ER-F1 to F5) were identified as dairy farms, one (Lo-T1) as a trader associated to a dairy farm, one (Tr-T1) as a trading farm and one (ER-F2) as resting stable.

Regarding the Lombard outbreaks, epidemiological information led to the hypothesis of a connection between the cases of tuberculosis infection that emerged in the different years in this region. Such information revealed that probably this group of outbreaks have its origins in the positivity to *M. caprae* in the breeding farm/trader Lo-T1, isolated in 2002 from bovine ID 6627-2002. This farm, which was both a dairy farm and seller, had been building a solid distribution network since 2000, characterized by importing a high volume of animals from Austria and Germany and selling them to the local farms of Val Camonica, passing through the collection centres of Bolzano and Trento (Tr-T1, L, M, N). Like Lo-T1, I and J were also identified as traders belonging to the same Italian distribution network, but unfortunately, we did not have any positive samples from them, since Italian collection centres are excluded from diagnostic checks by the national bTB control and eradication program.

Starting from this first case, numerous outbreaks were detected in the provinces of Brescia and Bergamo (Lombardy), all with a different epidemiological distance to Val Camonica (Lombardy) traders (samples from Farm Lo-F1, Farm Lo-F2, Farm Lo-F3, Farm Lo-F4, Farm Lo-F5, Farm Lo-F6and Farm Lo-F7). In the subsequent years, in 2010 and 2015 other outbreaks emerged in Lombardy and the epidemiological investigation revealed that they were related to the previous ones, with Farm Lo-F8 and Farm Lo-F9 involved. Even more recently, in 2022, new outbreaks appeared, still linked to the above-mentioned including Farm Lo-F13, Farm Lo-F8 (already detected positive in 2010) and a positive calf sited in Farm Ve-F1, born from a cow from Farm Lo-F8. Three more outbreaks were found in 2018 out of the Val Camonica area, involving Farms Lo-F10, Lo-F11 and Lo-F12. Farm Lo-F11 and Lo-F12 were in the Valtellina area, Sondrio province, Lombardy, and Farm Lo-F10, located in the Basso-Iseo area, Brescia province, Lombardy.

The outbreaks of Emilia-Romagna region are shown in purple in Figure 2: farm ER-F1, ER-F3, ER-F4 and Farm ER-F5 are oriented towards dairy production, while ER-F2 is resting stable. The Farms ER-F1, ER-F2 and ER-F4 bought animals directly from Trentino Alto Adige traders (N, M, L, respectively). ER-F2 and ER-F4 had contact with the farms ER-F3 and ER-F5, respectively.

### WGS data analysis

All Italian samples were sequenced showing an average length of coverage of 4 286 069 bp (range 4 284 612- 4 286 484 bp), a GC content of 65.62% and a mean sequencing coverage of 146X (min 68X - max 270X). Austrian samples show an average length of 4 297 354 bp (range min 4 295 075 - max 4 300 718), a GC content of 65.63% and a mean sequencing coverage of 60X (min 47X - max 73X). Spoligotype SB0418 was in-silico confirmed for all isolates, while SNP analysis quantified pairwise distances among *M. caprae* isolates of this dataset (statistics result from MTBseq pipeline are reported in Additional file 4A for the Italian samples and Additional file 4B for the European samples).

Our samples, as well as those downloaded from SRA and those provided by AGES, were analyzed for the characterization of the RD4 region. All our samples and those of AGES were classified as the Lechtal subgroup (Additional file 7), while the others belonged to all three *M. caprae* subgroups [35, 60] as specified in Additional file 2.

### Phylogenetic analysis

**Figure 3:**
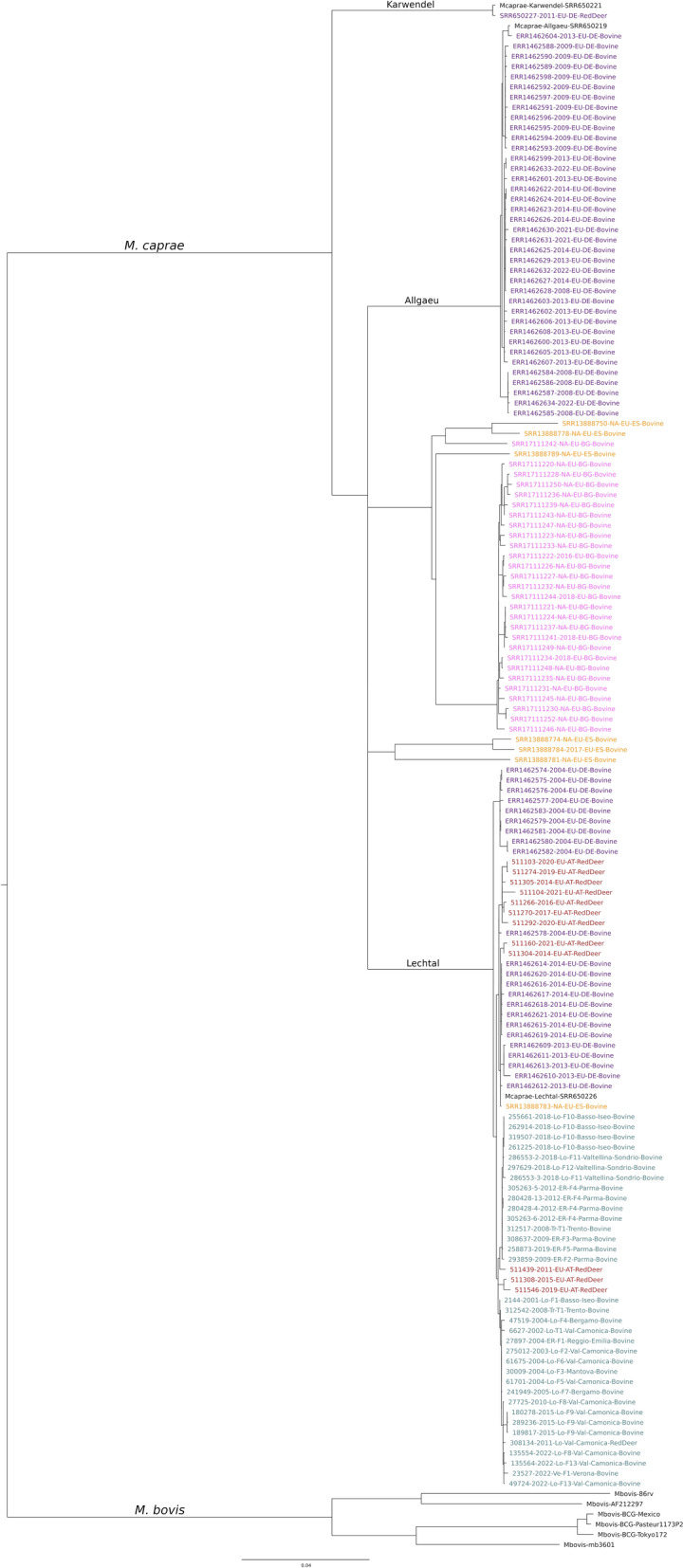
**Phylogenetic tree constructed on sequences of** ***M. caprae*** **strain SB0418 included in this study (cadet blue), Austrian sequences provided by AGES (red) and those available in public databases (Colour coded: German strains in purple, Bulgaria strains in pink, Spanish strains in orange and *M. bovis*** **reference strains in black). **The evolutionary history was inferred using the Maximum Likelihood Method, using the transversion model with empirical base frequencies (TVM + F) with a bootstrap of 1000X. Evolutionary analyses were conducted in IqTree2.

The phylogeny (Figure 3) constructed among the “European Dataset” confirmed that the Italian *M. caprae* strains described in this study clustered with the Lechtal subgroup, supporting previous experimental findings, clustering with all Austrian, some German sequences and a Spanish one. Particularly, three Austrian sequences figure within the Italian cluster.

All sequences were divided into four main clusters, linkable to the three main subgroups obtained by RD4 analysis, Lechtal, Allagau and Karwendel, plus a new cluster, made of Bulgarian and Spanish sequences (Figure 3). This separation pattern was confirmed also by the TBgroup analysis set with a 12 SNP threshold.

If we consider the pairwise-SNP of the European dataset (Additional file 5), our samples had a mean of 19.9 SNP (min 9, max of 37) compared to the other strains of the considered dataset belonging to the Lechtal subgroup. Our samples had a common ancestor with European strains belonging to the Lechtal subgroup isolated in Germany and Austria and they were closer phylogenetically (min 10 and max 27 SNP pairwise) with three Austrian strains isolated from red deer in 2011, 2015 and 2019. As expected, the nucleotide differences between our strains and Allgäu and Karwendel subgroups were higher (min 317 - max 395 and min 366 - max 400 SNPs, respectively).

**Figure 4:**
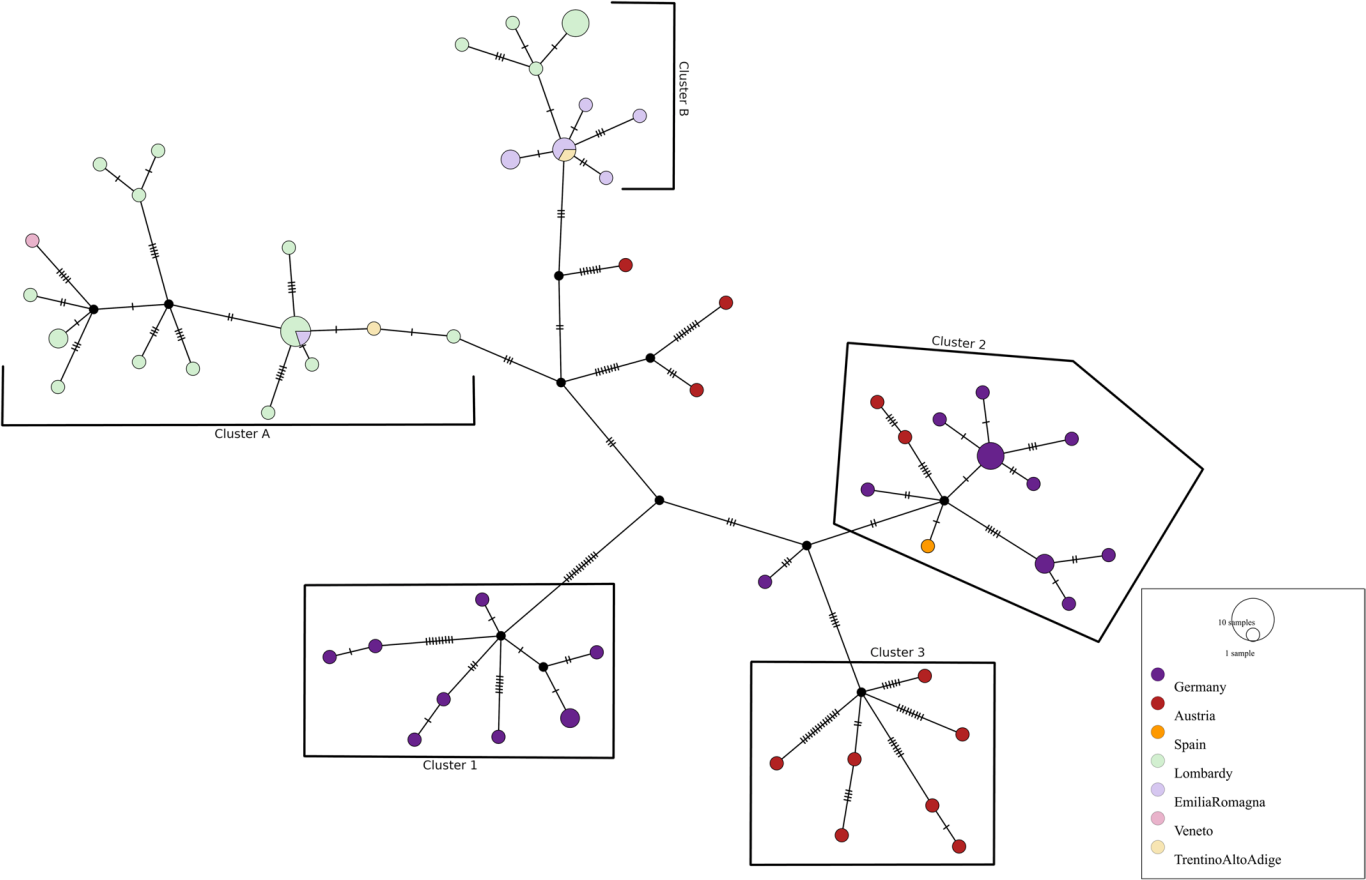
**Median-joining network based on SNP data of *M. caprae* genomes belonging to Lechtal cluster of Figure** **3****.** Dots are colour coded: green for the Lombardy region, violet for the Emilia-Romagna region, ochre for the Trentino region, pink for the only Veneto farm, red for Austrian sequences, purple for German sequences and orange for the Spanish sequence. The number of hatch marks represents the genetic distance (SNP) separating sequences with every hatch mark corresponding to a SNP. The size of the circle is proportional to the number of isolates sharing the same SNP-profile. Hypothetical genotypes are displayed as black circles to connect the genome sequences.

**Figure 5:**
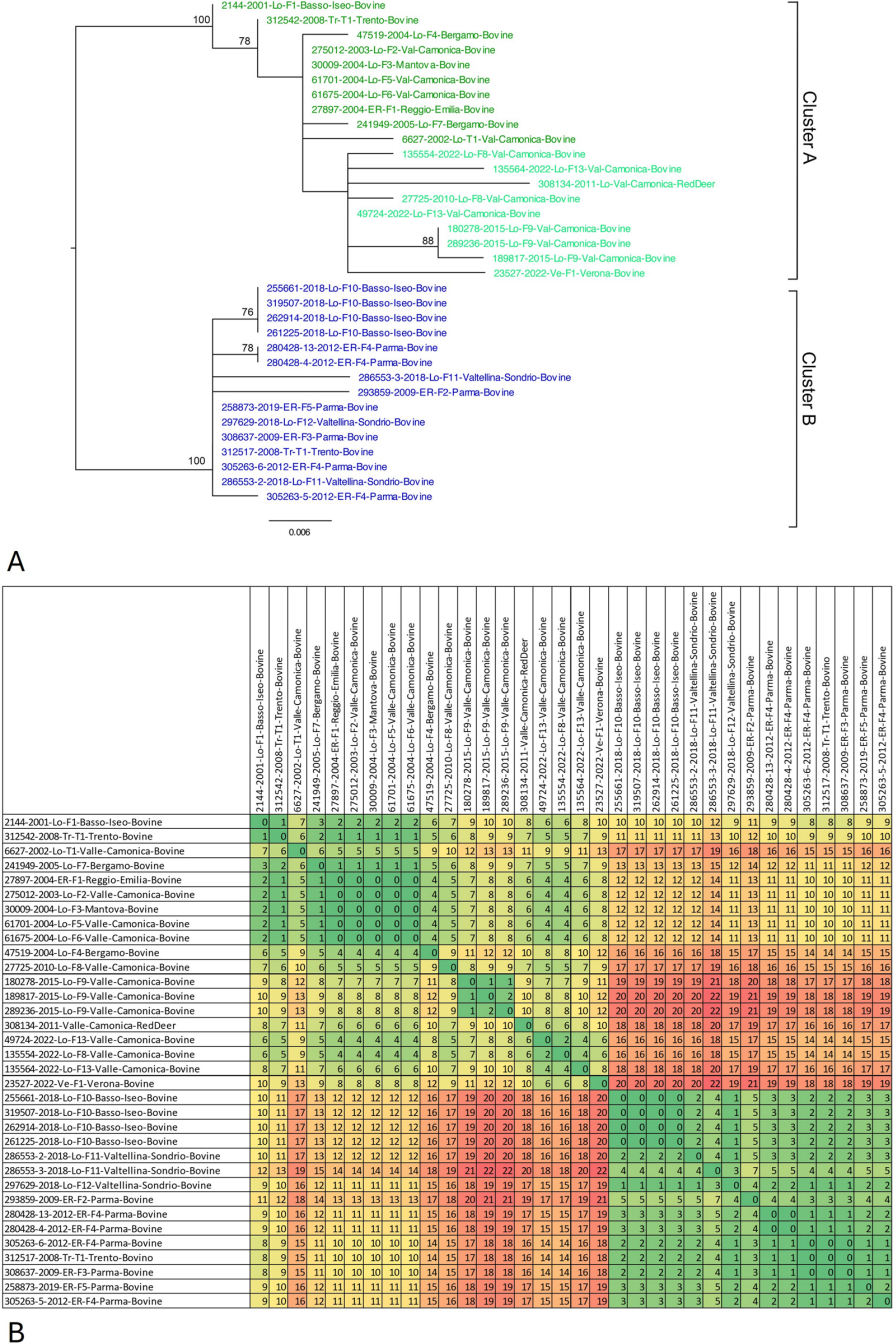
**Phylogenetic tree with respective distances matrix of Italian samples. A** Phylogenetic tree constructed on complete genomes of the considered Italian dataset. The evolutionary history was inferred using the maximum likelihood method, using the K2P model with a bootstrap of 1000. Colour code: cluster A in green comprising Lombardy isolates; cluster B in blue, comprising both Emilian and Lombard isolates. Light green indicates a particular branch of the tree. **B** DistanceSNP-matrix obtained from the alignment related to the tree. Colour code: greenish colours indicate fewer SNP differences, reddish colours indicate greater SNP differences.

A further analysis was conducted on the Lechtal cluster, comprising German, Austrian, Spanish and Italian samples: the median-joining network based on SNP data (Figure 4) allows to better visualize the mutations accumulated by the strains in the dataset and to suppose possible routes of transmission of *M. caprae* among countries and herds. Analysing the MJN obtained from the Lechtal-only tree, five different clusters were highlightable, with an unsampled common ancestor in the middle. Cluster number one comprises only German bovines and cluster number three comprises only Austrian red deer samples, both with unsampled ancestors in common. The second cluster comprises both German bovines, Austrian red deer and the Spanish bovine, with an unsampled ancestor in common. Italian samples were divided into two different clusters, named A and B with three Austrian red deer and an unsampled common ancestor. Between Italian sequences and unsampled common ancestor, few SNP were detected (*n* = 3), while a greater number of SNP was calculated considering Austrian red deer samples. Italian sequences are furthermore analysed below, as shown in Figure 5A with the phylogenetic tree made of Italian samples, and Figure 5B with the distance SNP matrix obtained by this alignment.

The inferred phylogeny, performed on Italian samples only, identified two clusters (A and B) with a between-cluster pairwise SNP range of 6-16 SNP.

Cluster A (the green one in Figure 5A) comprises isolates mainly from the Lombardy region (pairwise SNP range = 0–7 SNP) and is further divided following a temporal distribution; genomes closest to the root are the oldest ones (coloured in dark green in Figure 5A), while the most recent genomes are comprised in a branch of the tree, coloured in light green in Figure 5A. In particular, the first ones were isolated in Val Camonica (2144-2001, 6627-2002, 275012-2003, 61675-2004, 61701-2004, 27897-2004), Bergamo (47519-2004 and 241949-2005) and Mantua provinces (30009-2004) in 2001–2005, and share a pairwise SNP distance from 0 to 4 SNP while the more recent branch includes samples isolated in 2010–2022, and the red-deer sample [40], with 0—7 SNP identified. Three extra-Lombardy cases fell in cluster A: 312542-2008, 23527-2022 and 27897-2004.

Cluster B (the blue one in Figure 4) includes isolates with a pairwise SNP range of 0-6 SNP and it is composed of strains collected from farms from Emilia- Romagna from 2008 to 2019 (Farm ER-F2, ER-F3, ER-F4, ER-F5), sample 312517-2008, second isolates from the Trader Tr-T1 of Trentino Alto Adige (pairwise SNPs: 0-5 SNPs), and 7 strains isolated from Lombardy farms Lo-F11 (286553-2-2018 and 286553-3-2018), Lo-F10 (255661-2018, 262914-2018, 319507-2018, 261225-2018) and Lo-F12 (297629-2018).

After testing all possible TB group thresholds, from 5 to 12 SNP, the only threshold leading to the formation of two distinct groups was 5, which perfectly aligned with the available epidemiological data; on the contrary thresholds from 6 to12 SNP did not result in a grouping of the sequences (Additional file 6), as all taxa were clustered together, regardless of the epidemiological links identified.

## Discussion

Bovine tuberculosis is a significant concern due to its health implications and economic impact, and an integrated approach that combines epidemiology and molecular biology can support eradication plans by tracing both the origin and routes of transmission of bTB outbreaks.

*M. caprae* is mainly known in the European context, where it has been described in several species; its complex epidemiology, similar to that of *M. bovis* [13], proved the difficulties in eradicating bTB.

Despite being declared Officially Bovine Tuberculosis Free (OTF) since 1999 and since 1996, Austria and Germany have reported cases of infection, both in livestock and wildlife [7, 22, 23, 35, 36, 60–62].

In Austria, *M. caprae* has been identified as a sporadic pathogen in the western regions, where dairy farming is prevalent [62]. The disease’s persistence is linked to both traditional farming practices and the interaction between cattle and red deer, recognized as wildlife reservoirs in these areas, particularly where grazing lands overlap [36, 62]. Studies and surveillance data indicate that the infection rates in deer can influence the dynamics of tuberculosis in both wildlife and adjacent livestock communities, considering also the bTB high prevalence (up to 23%) found in a specific red deer population back in 2011 in the Southern part of the District of Reutte [62, 63]. Numbers of tested animals and cases of *M. caprae* are regularly published in official reports and on the webpage of AGES [64]. As in many countries, an intensive control program for cattle is being implemented: a rigorous test-and-cull scheme is in place to early detect any transmission from this reservoir to livestock and prevent any further cattle-to-cattle spread between farms. Tracing is performed for each case in cattle.

In Germany, especially in the Bavaria region, *M. caprae* has been more frequently identified in wildlife, suggesting a wildlife-livestock transmission cycle. The German approach relies on surveillance, with an emphasis on identifying and managing wildlife reservoirs. Genetic studies with WGS have been used for tracing transmission and understanding the molecular epidemiology of the disease within and across borders [28, 65, 66].

Switzerland, from which 312517-2008 bovine was imported, has been declared OTF since 1960, reporting only a few *M. caprae* outbreaks in 2013-2014, 2019 and 2023 [67].

In Italy, the bTB epidemiological situation is different among regions: while most of the northern and central regions acquired OTF status during the past years (e.g. Lombardy in 2010, Veneto region in 2008, Emilia-Romagna Region in 2007, Trento and Bolzano in 2003), many southern regions are still struggling with the disease.

The number of infection cases caused by *M. caprae* is much lower compared to those caused by *M. bovis* [45] and most *M. caprae* outbreaks are currently located in southern Italy. Despite this, in the northern regions two different introduction routes have been identified in the past years, resulting in a higher number of outbreaks in the north associated with the genotype analysed in this study. Notably, *M. caprae* isolates from these northern outbreaks belong to the Lechtal subgroup. Fink et al. [36] clarified the prevalence of the disease among red deer in the alpine region arc: out of 514 sampled red deer in Italian territories, only one was positive to bTB, as documented by Chiari et al. [48]. Wildlife management practices and hunting rules introduced [48, 62, 68], in addition to the natural barrier represented by the Alps, are the main reason behind the low prevalence of *M. caprae* among wildlife, particularly red deer. Following this project, despite the absence of a national wildlife monitoring plan, all red deer remains are inspected by trained personnel, in order to identify lesions rapidly. Furthermore, in Italy, WGS-based studies, able to finely discriminate the strains and to support the identification of the transmission routes of the disease in the examined territory, are still missing.

In this study, we aimed to investigate a series of outbreaks caused by *M. caprae* that occurred in bovine herds in a limited area of northern Italy between 2001 and 2022 using classical epidemiology data and WGS. This approach aims to provide an initial understanding of the strain’s molecular diversity and transmission patterns starting from the available epidemiological information. Indeed, as already reported by others [55], when epidemiological information is not available, MIRU-VNTR analysis is still considered a strong and reliable tool to select samples to be included in an infection tracing study.

Starting from the European scenario, all the Italian and Austrian samples were confirmed to belong to the Lechtal subgroup. The European isolate analysis shows that, except for Bulgarian and some Spanish sequences, all other isolates clustered within the three subgroups defined by the RD4 region. The sequences forming a distinct stand-alone cluster may reflect a divergent deletion pattern in their RD4 region.

The analysis, based only on this genotype, confirms a connection between German, Austrian and Spanish, and Italian and Austrian samples, inferring common unsampled ancestors.

From this analysis, it is also possible to highlight the same distribution among clusters for Italian sequences, which was obtained in the Italian sequences tree. The phylogenetic analysis highlighted how our samples are distributed into two clusters, differing from each other by 6-16 SNP. Interestingly, they correspond to the sample spatial separation between the two regions involved, suggesting an independent entry of the infection into the two regions from the same origin.

The interpretation of genomic results in light of the contact tracing data suggests a possible route of transmission that starts from Austria/Germany, goes through Trentino Alto Adige traders, and lands in Val Camonica (Lombardy) and Emilia Romagna by two distinct routes.

Once the infection reached the Italian territory, it spread to other farms in various ways, like sharing pastures, contacts between farmers, buying and selling animals, and animal movements.

It is also important to consider a particular management practice spread among Lombardy valley farmers, which consists of the temporary transfer of their animals to other farms, where a hay-based diet is provided, from the end of the summer until the end of the winter, collecting animals from different farms in the same place. Unfortunately, the epidemiological investigation did not report any information about these exchanges, but this practice cannot be ignored since it could have played an important role in facilitating the transmission of bTB between animals of different farms.

The presence of two clusters (A and B) is strongly reliable, due to their similarity (max 16 SNP), supported by a hypothetical ancestral strain that might be in Austria or Germany. The infection could have spread through traders in Trentino Alto Adige and then reached farms of the territory of Lombardy and Emilia-Romagna, in different and independent ways.

In Lombardy, the infection was introduced by local sellers of Val Camonica (Lo-T1, I, J) in the early 2000 (the first isolate was in 2001). These sellers, who travelled to local farms to sell animals, caused the emergence of the first cases of 2002 and 2005, which are grouped at the cluster root, resulting in the sampled sequences 6627-2002 (Lo-T1), 47519-2004 (Lo-F4) and 241949-2005 (Lo-F7). The infection later appeared in other farms between 2010 and 2015 and it was detected again in 2022, showing additional SNP (1-3). Starting from the tree topology and matching epidemiological information, the light green branch of the tree, highlighted in Figure 5a, seems to originate from a more recent isolate (49724-2022). The epidemiological investigation revealed that 2022 isolates (135554-2022, 135564-2022 and 49724-2022) were collected from older bovines (Additional Table 1), which in 2010, entered in contact with other animals involved in a bTB outbreak. The tree topology suggests a genetic drift of subtle changes between isolates, resulting in a consecutive flow of outbreaks, related to each other.

In Emilia Romagna farms (Cluster B), the epidemiological investigation highlighted individual contacts between the farms and the Trentino Alto Adige Traders (ER-F2 related to M and ER-F4 related to L), which seem to be the source of the Emilian outbreaks. The strains responsible for the infections in the interested farms were very similar (0-6 SNPs of difference) and this evidence suggested that they were correlated with each other. However, it is difficult to delineate a unique pathway of infection. The presence of samples from Lombardy in the Emilia Romagna cluster highlighted a potential scenario where the bovine ID 286553-2-2018 was bought by the farm Lo-F11 directly from Germany, possibly already infected. Furthermore, the owner of this farm was related to the manager of the Lo-F10 farm, facilitating animal exchanges, probably causing a further spread of TB infection. Farm Lo-F12 was geographically close to farm Lo-F11 and the authorities hypothesized that infection occurred through contaminated sewage spreading by Farm Lo-F11 in common areas.

Other than this scenario, three specific situations need further clarification:

•312542-1-2008 is from Trader T1 (Trento province, Trentino Alto Adige region), one of the traders that bought animals from Germany and sold them to traders of Val Camonica. These samples grouped at the root of the A cluster with the older isolates of the same area.
•Sample 27897-2004 is from Farm ER-F1 of Reggio Emilia (Emilia Romagna region). This farm did not have any evident exchange and direct connection with the samples of Val Camonica. However, the contact tracing highlighted that its infected bovine was bought from an unknown trader of Trento, who might be the same trader of the Val Camonica area. Additionally, the ER-F1 farmer bought animals directly from Germany and Austria, indicating a potential common origin of the infection between this case and those of the Lombardy region.
•Sample 23527-2022 is from a calf from the Ve-F1 Farm in Verona province, Veneto region, but it is in cluster A, along with Lo-F8 sequences. This can be explained with available epidemiological information since this calf originated from the Farm Lo-F8 of Val Camonica (Lombardy), and was born from the cow 135554-2022, located in the same cluster.

Interestingly, the clustering of the red deer sequence with the strains isolated from bovines in Val Camonica suggested the involvement of wildlife in the same infection cluster, likely due to shared grazing areas, confirming what had already been reported by various authors in Tyrolean and Bavarian Alps [36, 62].

Analysing epidemiological data (Figure 2), animal exchanges (arrows) and pasture sharing (p) play a crucial role in *M. caprae* spread. Potential sources of indirect infection, like the use of contaminated vehicles for animal transportation (dotted black lines) or the spread of contaminated sewage between close farms (dotted coloured lines), could have acted as an indirect way of disease spreading through farms. It should be noted that in Italy, a health check is performed during animal movements between farms only if non-OTF territories are involved in the exchange: local health authorities’ veterinary services carry out the intradermal tuberculin test during the 30 days before or after the introduction into the farm.

The extreme genetic homogeneity of *M. caprae*, like other members of the MTBC (0.15-0.5 substitutions per genome per year) [13, 69, 70], makes the finer scale disease tracing of outbreaks in the short temporal window not feasible. In such settings, as described by many authors, genomic data from MTBC organisms and Bayesian inference can have relatively limited utility [41, 71–74]. So, phylogenesis is useful to understand how a particular case/outbreak is related to other events, but we should also consider the robustness of the analysis to avoid wrong deductions.

Starting from the assumption that evolution is a stepwise accumulation of forward mutations, we can infer the possible direction of transmission from SNP analysis obtained via WGS data. In particular, the connections reported by the median-joining network analysis (Figure 4) suggest a readable and simplified representation of the number of SNP between samples and can lead to build a hypothesis of transmission with the addition of a presumptive common source. Regarding the MJN, the smaller number of SNP suggests a very close relationship between Italian and unsampled ancestors, shared with Austrian sequences: this might be related to the specific animal importation documented by epidemiological investigations, while the greater number of SNP enhanced with Austrian red deer samples might be ascribable to older events [75, 76].

In this work, we evaluated, for the first time in Italy, *M. caprae* outbreaks using WGS data, finding the most suitable SNP threshold, according to our specific dataset. Generally, a 5 SNP cut-off has been widely accepted for clustering of recently linked cases, while 12 SNP incorporates also older transmission events [55, 66, 76–78]. By testing different thresholds (ranging from 5 to 12 SNP), we determined that a cut-off of 5 SNP is the optimal parameter for this scenario. With this threshold, 2 groups were highlighted, corresponding to the Cluster A and B, which align with the epidemiological data. Our results were in line with other previous studies conducted in Europe [28, 75, 79, 80]; however, the ideal threshold must be determined based on the specific epidemiological context of each setting, including more studies and increasing sample number.

*M. caprae* infections are raising concern, widening their geographical range and consolidating their presence among both livestock and wildlife. Considering their potential economic impact, it is important to implement control measures that make use of both field and molecular activities.

This work underlines the crucial role of WGS and analysis of single nucleotide polymorphisms in *M. bovis*/*M. caprae* as robust and reliable methods for either supporting or refuting hypotheses arising from classical epidemiological investigations. It provides objective data for establishing outbreak connections and tracing infections, particularly in contexts where metadata are limited or incomplete, and consolidates the acceptable SNP threshold scenario delineated not only in the national Italian context, but also in the European framework.

It comes to light that the analysed strains have a high genetic similarity and share a common origin, as characterized by low SNP numbers. The comprehensive genome sequencing approach provides essential data for confirming connections between the involved farms and objectively validated hypotheses derived from epidemiological investigations, originally based solely on contact tracing.

## Supplementary Information

**Additional file 1. List of Italian Samples considered in this article.**

**Additional file 2. List and relative metadata of genomes included in General, European and Lechtal Dataset, used for phylogenetic analysis.**

**Additional file 3. Epidemiological connections between Italian samples.**

**Additional file 4. MTBseq Statistic output of Italian and European Samples.**

**Additional file 5. European dataset tree SNP table.**

**Additional file 6. MTBseq Groups output with different Distances parameter set.**

**Additional file 7. IGV representation of the three different *M. caprae* genotypes.**

## Data Availability

The obtained sequences are available in NCBI BioProject PRJNA1123928, with SRA accession number from SRR29410356 to SRR29410390 and BioProject PRJNA1173331 with SRA accession number from SRR31790111 to SRR31790122.
