# Supplementary figures and images for: M. caprae in northern Italy: a comprehensive analysis through whole-genome sequencing on the genetic variability in bovine herds
Source: Vet Res. 2025 Aug 7;56:163. doi: 10.1186/s13567-025-01585-x (PMC12330137; doi:10.1186/s13567-025-01585-x)

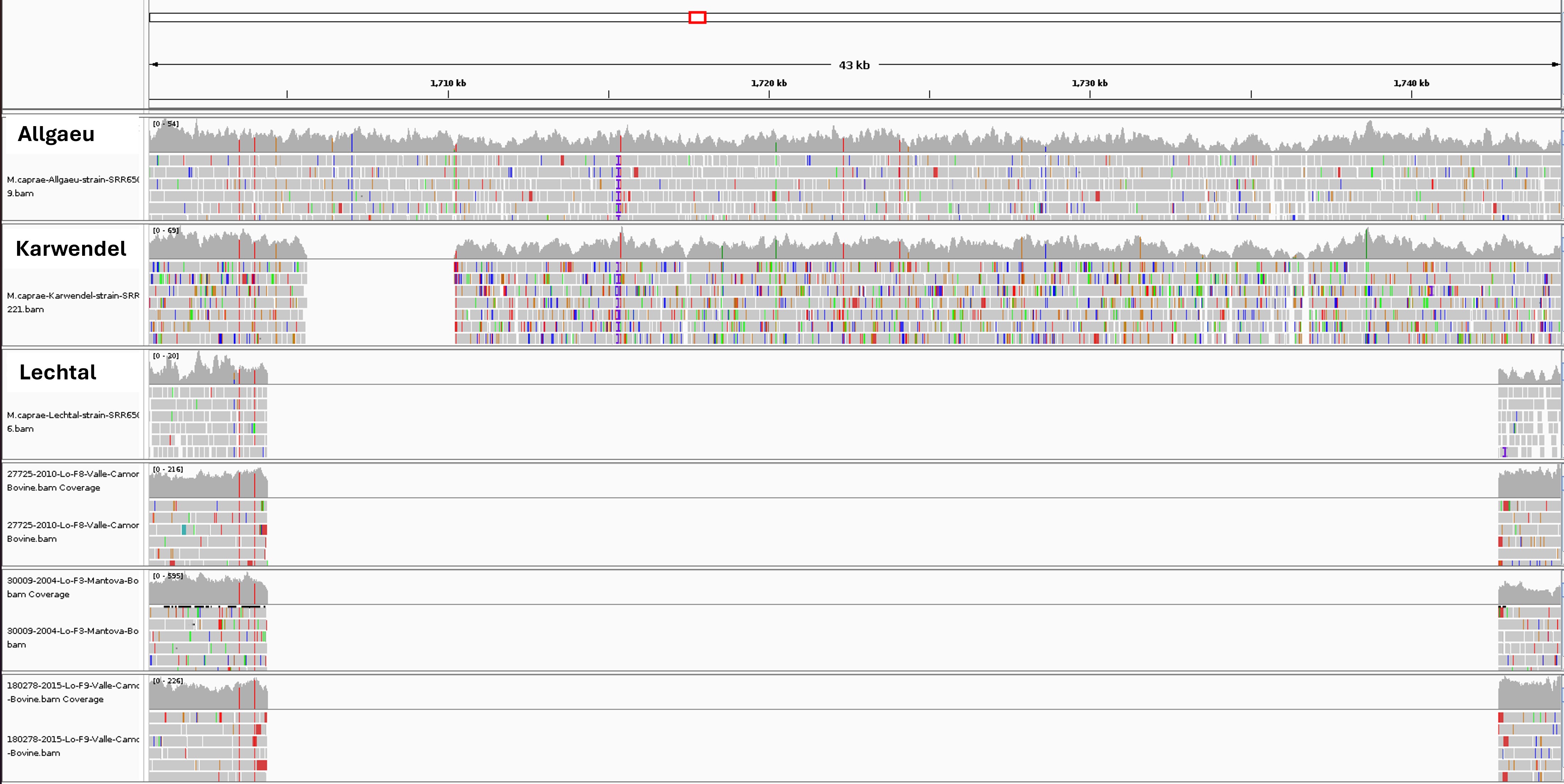

Supplement: Supplementary file 7 — Additional file 7. IGV representation of the three different M. caprae genotypes. [file 13567_2025_1585_MOESM7_ESM.jpg]
